# Thalamocortical-auditory network alterations following cuprizone‐induced demyelination

**DOI:** 10.1186/s12974-016-0629-0

**Published:** 2016-06-22

**Authors:** Nikoo Ghaffarian, Masoud Mesgari, Manuela Cerina, Kerstin Göbel, Thomas Budde, Erwin-Josef Speckmann, Sven G. Meuth, Ali Gorji

**Affiliations:** Epilepsy Research Center, Westfälische Wilhelms-Universität Münster, University of Münster, Robert-Koch-Straße 27a, 48149 Münster, Germany; Department of Neurology, Westfälische Wilhelms-Universität, University of Münster, Albert-Schweitzer-Campus 1, Building A1, 48149 Münster, Germany; Institute of Physiology I, Westfälische Wilhelms-Universität, Münster, Germany; Department of Neurosurgery, Westfälische Wilhelms-Universität Münster, Münster, Germany; Shefa Neuroscience Research Center, Khatam-Alanbia Hospital, Tehran, Iran

**Keywords:** Auditory cortex, Internal capsule, Neuronal activity, Long-term potentiation

## Abstract

**Background:**

Demyelination and remyelination are common pathological processes in many neurological disorders, including multiple sclerosis (MS). Clinical evidence suggests extensive involvement of the thalamocortical (TC) system in patients suffering from MS.

**Methods:**

Using murine brain slices of the primary auditory cortex, we investigated the functional consequences of cuprizone-induced de- and remyelination on neuronal activity and auditory TC synaptic transmission in vitro.

**Results:**

Our results revealed an impact of myelin loss and restoration on intrinsic cellular firing patterns, synaptic transmission, and neuronal plasticity in layer 3 and 4 neurons of the auditory TC network. While there was a complex hyper- and depolarizing shift of the resting membrane potential, spontaneous and induced action potential firing was reduced during demyelination and early remyelination. In addition, excitatory postsynaptic potential amplitudes were decreased and induction of LTP was reduced during demyelination.

**Conclusions:**

These data indicate that demyelination-induced impairment of neurons and network activity within the TC system may underlie clinical symptoms observed in demyelinating diseases, corroborating human findings that disease progression is significantly correlated with microstructural tissue damage of the TC system. Further investigation into focal inflammation-induced demyelination models *ex vivo* and in vivo are needed to understand the functional implication of local and remote lesion formation on TC network activity in MS.

## Background

Multiple sclerosis (MS) is a chronic inflammatory demyelinating disease in which myelin sheaths wrapping the axons in the brain and spinal cord are damaged [1]. The symptoms of the majority of patients with MS usually begin with alternating periods of neurological disability and recovery that can last for many years. Slowly expanding demyelination accompanied by axonal damage and neuronal degeneration is well-accepted pathological hallmarks of neurological deficits in MS, and mechanisms resulting in remyelination of axons are thought to be important for restoration of neuronal function following relapses [2].

Myelin loss affects the integrity of neuronal networks and their synaptic plasticity [3]. Abnormalities in the spontaneous firing patterns of neurons have been reported in both in vitro and in vivo models of peripheral demyelinated axons [4–6]. In the central nervous system (CNS), myelin loss was demonstrated to cause highly heterogeneous alterations in nodes of Ranvier, thereby affecting the excitability of neocortical pyramidal neurons [7]. Scarce data exist on the effect of remyelination on the neuronal network activities. In addition to neuronal damage, demyelination induced abnormal patterns of cortical synaptic plasticity in a group of MS patients [8] and in different animal models of demyelination [9]. Enhancement of synaptic plasticity in the motor cortex due to the alteration of the functional properties of surviving neuronal circuits has been suggested as a crucial factor for recovery from relapse-associated neuronal damage in MS patients [10].

The auditory thalamocortical pathway is the only neural substrate that sends precise frequency information to the auditory cortex [11]. Impaired thalamocortical auditory connectivity underlies the functional abnormalities in several neurological disorders, such as epilepsy, autism, and schizophrenia [12–14]. Increasing evidence suggests extensive involvement of the thalamocortical pathway in patients with MS [15]. Any disruption in this topographically organized circuit and distortions in the temporal processing of frequency information may trigger the symptoms observed in MS [16, 17]. Indeed, the myelin sheath breakdown along the auditory pathway was accompanied by abnormalities of axonal conduction and auditory deficits in MS patients [18]. Abnormalities in auditory evoked potentials and the cognitive P300 wave indicated dysfunction of different regions of the central auditory pathway and pointed to a predictive value of different evoked potentials [19, 20]. Several toxins have been used to produce demyelination in animals, including cuprizone. Feeding of cuprizone, a copper-chelating mitochondrial toxin, induces oligodendrocyte injury and demyelination in different brain regions, preferentially in the corpus callosum. Cuprizone-induced demyelination is usually followed by complete remyelination within a few weeks. Thus, cuprizone treatment serves a useful animal model for investigation of the processes involved in demyelination and consequent remyelination [21–23]. To study how demyelination may affect neuronal function and synaptic transmission of the thalamocortical system, we used the cuprizone-mediated demyelination/remyelination model to investigate single cortical neuron properties as well as synaptic plasticity under demyelinated and remyelinated conditions in the auditory cortex in vitro.

## Methods

### Animals

Thirty-seven C57BL6 mice (8–12 weeks old) were group-housed (four animals per cage) under a constant temperature (20–22 °C) and 12-h lighting cycle, and during this time, food pellets and water were available ad libitum. All experiments were conducted according to the guiding principles for the care and use of animals in the University of Münster, Germany (LANUV approval AZ 87-51.04.2010.A331).

### Experimental design

Mice were divided randomly into four groups: (*i*) control group which received normal powdered chow for 5–6 weeks (*n* = 6), (*ii*) cuprizone group that was fed powdered chow mixed with 0.2 % cuprizone for 5–6 weeks (*n* = 9), (*iii*) 7 days remyelination group that received normal diet for 7 days after 5–6 weeks of cuprizone diet (*n* = 11), and (*iv*) 25 days remyelination group which received normal diet for 25 days after 5–6 weeks of cuprizone diet (*n* = 11). All mice from different groups were sex- and age-matched [24].

### Slice preparation

The experiments were carried out on brain slices (500 μm) containing the primary auditory cortex (A1), the thalamic medial geniculate nucleus, the lateral geniculate nucleus, the nucleus reticularis thalami, the ventrobasal nuclear complex, and functional thalamocortical projections. Slices were prepared as previously described [25]. Briefly, the brain was removed under deep isoflurane anesthesia (Abbott, Wiesbaden, Germany). Brains were glued with the dorsal side onto a 25° agar-ramp and then were placed into a vibratome and superfused with 4 °C artificial cerebrospinal fluid (ACSF). The ACSF contained (in mmol/l): NaCl 124, KCl 4, CaCl_2_ 1.0, NaH_2_PO_4_ 1.24, MgSo_4_ 1.3, NaHCO_3_ 26, and glucose 10. The ACSF was continuously equilibrated with 5 % CO_2_ in O_2_, stabilizing the pH at 7.35–7.4. Slices were pre-incubated at 28 °C for 60 min in ACSF. After 30 min pre-incubation, CaCl_2_ was elevated to 2.0 mmol/l. Thereafter, slices were transferred to an interface recording chamber and superperfused with ACSF at 32 °C.

### Electrophysiological recordings

Intracellular recordings were performed in the layer 4 of the primary auditory cortex (A1); using sharp microelectrodes filled with 2 mol/l potassium methylsulphate (connected to a 2 mol/l KCl solution-bridge through a ceramic junction; 60–100 MΩ, 32 °C). We used sharp electrodes instead of whole-cell patch-clamp electrodes to diminish the alteration of the intracellular compartment as far as possible. After impaling, neurons revealed frequent periods of spontaneous action potential (AP) firing. A stable resting membrane potential (RMP) ranging from −48 to −63 mV was determined during periods when neurons were not spontaneously active. A constant positive or negative current was injected into individual neurons to set the membrane potential to −40 or −70 mV. The reference electrode and the connection to the microelectrodes were symmetric silver-silver chloride bridges. Intracellular current pulses were passed via an active bridge circuit, and bridge balance was monitored and adjusted during intracellular recordings. The following criteria were considered for the inclusion of data in the study: (*i*) recording stability without any sign of injury discharges and (*ii*) RMP more negative than −45 mV with deviation less than 5 % during the control recording period [26]. A bipolar extracellular stimulating electrode was placed in the thalamocortical projections in rostral position with respect to the cortex and inhibitory and excitatory postsynaptic potentials (IPSPs and EPSPs) were evoked in the primary auditory cortex (see inset in Fig. 4). The traces were digitized by a Digidata 1200 (Axon Instruments, CA, USA), and the data were collected and analyzed by Axoscope 10 (Axon Instruments, CA, USA).

The amplitude of APs was measured from RMP baseline to peak. The duration of action potentials was measured as the half-amplitude width. The amplitude of afterhyperpolarizations (AHP) of APs was measured from RMP to the peak of deflection [27].

### LTP

Electrical stimulations were applied via a bipolar platinum electrode attached to the thalamocortical projections in rostral position in respect to the cortex (see inset in Fig. 5). Using extracellular glass microelectrodes (150 mmol/l NaCl; 2–10 MΩ), extracellular local field potentials were recorded in the third layer of the auditory neocortical slices. Stimulation intensity was decreased to bring the evoked field excitatory postsynaptic potential (fEPSP) amplitude to approximately 50 % of maximum amplitude. In long-term potentiation (LTP) experiments, the auditory cortex was repeatedly stimulated once every minute. The tetanic stimulation consisted of a 100-ms long train of 100-Hz electrical pulses. The duration of the electrical pulse was 0.1 ms with an interpulse interval of 10 ms. Four trains were delivered to the thalamocortical projections with a train interval of 100 ms. LTP was described as the mean alteration in fEPSP amplitude (measured from the most positive to the most negative deflection) in response to five stimuli given 30 min after tetanic stimulation compared to the mean response to five pulses applied immediately before tetanic stimulation. fEPSP recordings were monitored and analyzed using WinLTP [28].

### Immunohistopathological studies

Immunohistochemistry was performed for each time point, namely week 0 for control, week 6 for demyelination, and then at 7 and 25 days of remyelination. Briefly, mice were perfused with 4 % paraformaldehyde (PFA) in phosphate buffer via the left cardiac ventricle as previously described [29]. Brains were removed, postfixed in 4 % PFA, and paraffin-embedded. For light microscopy, 7-μm serial paraffin sections were cut and dried at 37 °C overnight, as described before [23]. Paraffin-embedded sections were de-waxed, rehydrated, and microwaved for 5 min in 10 mM citrate buffer (pH 6.0). Sections were quenched with H_2_O_2_, blocked for 1 h in phosphate-buffered solution containing 3 % normal goat serum, 0.1 % Triton X-100, and then incubated overnight with the primary antibody. The primary antibody for myelin proteolipid protein (PLP; mouse IgG2a, 1:500, Serotec) was used to detect de- and remyelination. After washing, sections were further incubated with biotinylated anti-mouse IgG (heavy and light chain) secondary antibodies (1:500, Vector Laboratories) for 1 h followed by peroxidase-coupled avidin-biotin complex (ABC Kit, Vector Laboratories). Reactivity was visualized with diamino-3,3′-benzidine (Vector Laboratories). For cell staining, slides were counterstained using Mayer’s hemalum solution (Merck). The extent of myelination was subsequently analyzed by light microscopy (Olympus BX61).

### Statistical analysis

All data are given as mean ± SEM. Multiple comparisons were performed using analysis of variance (ANOVA) test followed by the Dunn’s test. Significance was established when the *P* values were less than 0.05.

## Results

### The functional impact of de- and remyelination on cellular properties

In the auditory cortex, layer 4 neurons are the first cellular elements in signal processing where thalamic sensory information is integrated and directed to other cortical layers [30]. To study the intrinsic firing properties of these A1 neurons, intracellular recordings were performed in combined brain slices containing the preserved thalamocortical auditory pathway [25].

**Fig. 1:**
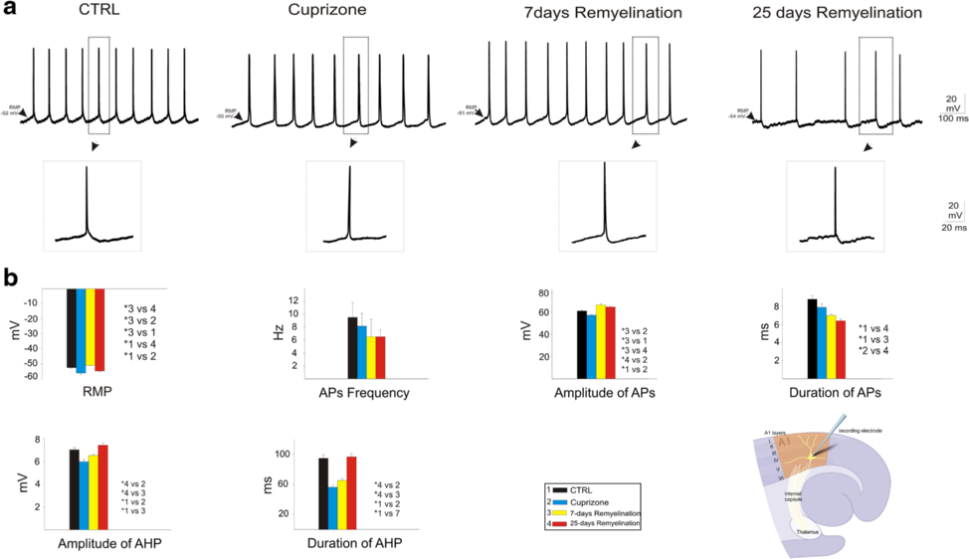
The membrane potential fluctuations recorded from layer 4 of the neocortical A1 area in control, cuprizone, 7 days remyelination, and 25 days remyelination mice groups. **a** Representative traces of the spontaneous membrane potential fluctuations in each mice group and a diagram of the thalamocortical slice, indicating recording site for intracellular recordings. **b** The resting membrane potentials (RMP), repetition rate of action potentials (APs), the amplitude and duration of APs, and afterhyperpolarizations (AHP) of APs in different mouse groups revealed the effect of demyelination and remyelination on different characteristic features of neuronal activities. *P < 0.001

Neurons from cuprizone-treated mice showed a significantly hyperpolarized RMP (−56.1 ± 0.8 mV) compared to control animals (−52.5 ± 0.4 mV; *P* < 0.001; Fig. 1a, b, Table 1). During the early phases of remyelination (7 days), neurons were characterized by a significantly more positive RMP (−51 ± 0.1 mV) compared to cuprizone and control mice (*P* < 0.001; Fig. 1a, b, Table 1). In the 25 days remyelination group, the RMP returned to a hyperpolarization state (−54.7 ± 0.4 mV), which was significantly lower than cuprizone and 7 days remyelination group (*P* < 0.001; Fig. 1a, b, Table 1). All neurons recorded revealed spontaneous AP firing with frequencies around 8 Hz. The duration of AHP of APs recorded from neurons in control mice as well as the 25 days remyelination group was significantly longer, and their amplitude was significantly higher compared to cuprizone-treated and 7 days remyelination mice (*P* < 0.001; Fig. 1a, b, Table 1). There were no significant differences in the repetition rate of APs between different groups (Fig. 1a, b, Table 1). Demyelination did not change the amplitude and duration of APs compared to control (Fig. 1a, Table 1). However, during the early and late phases of remyelination, the amplitude of APs significantly increased while their duration decreased (*P* < 0.001; Fig. 1a, b, Table 1). Demyelination as well as remyelination at both points in time revealed significantly reduced differences between prevailing membrane potential shortly before and the threshold potential of APs (*P* < 0.001; Fig. 1a, Table 1).

**Table 1:** Characteristics of membrane potential alteration in the primary auditory cortex in control, cuprizone, 7 days remyelination, and 25 days remyelination mouse groups

(A)							
Experimental groups	RMP (mV)	THP (mV)	APs_amp_ (mV)	APs_dur_ (ms)	AHP_amp_ (mV)	AHP_dur_ (ms)	APs frequency/s
CTRL (*n* = 17)	−52.5 ± 0.4	5.4 ± 0.2	60.9 ± 0.8	8.8 ± 0.4	7.1 ± 0.2	94.8 ± 5	9.5 ± 2.3
Cuprizone (*n* = 11)	−56.1 ± 0.8	4.6 ± 0.1	57.1 ± 0.6	7.9 ± 0.4	5.9 ± 0.2	56.1 ± 3.4	8.14 ± 2
7 days remyelination (*n* = 18)	−51.01 ± 0.1	4.5 ± 0.1	66.3 ± 0.4	7 ± 0.2	6.5 ± 0.1	65.3 ± 3.2	6.5 ± 2.7
25 days remyelination (*n* = 16)	−54.7 ± 0.4	4.1 ± 0.1	64.5 ± 1	6.4 ± 0.2	7.4 ± 0.2	96.9 ± 4.1	6.2 ± 1.1
(B)							
Hyperpolarization (RMP = −70)			THP (mV)	APs_amp_ (mV)	APs_dur_ (ms)	AHP_amp_ (mV)	AHP_dur_ (ms)
CTRL			5.4 ± 0.3	63.1 ± 1.1	8.9 ± 0.6	6.9 ± 0.3	158.9 ± 13.6
Cuprizone			3.9 ± 1.2	61.6 ± 0.3	9.9 ± 1.3	5.6 ± 0.3	65 ± 5.2
7 days remyelination			4.2 ± 0.2	65.6 ± 1.6	6.5 ± 0.6	4.6 ± 0.4	95.4 ± 10
25 days remyelination			4.6 ± 0.3	64.5 ± 3.8	8.5 ± 0.7	9.1 ± 0.4	139.4 ± 13.7
(C)							
Depolarization (RMP = −40)			THP (mV)	APs_amp_ (mV)	APs_dur_ (ms)	AHP_amp_ (mV)	AHP_dur_ (ms)
CTRL			4.5 ± 0.2	55.6 ± 1.2	6.6 ± 0.8	7.7 ± 0.2	94.4 ± 7.6
Cuprizone			4.1 ± 0.2	45.6 ± 2.1	6.9 ± 0.4	6.8 ± 0.3	54.1 ± 6.1
7 days remyelination			4.5 ± 0.1	53.6 ± 0.7	7.3 ± 0.3	6.3 ± 0.2	60.5 ± 4
25 days remyelination			4.4 ± 0.3	51.2 ± 1.3	8.9 ± 0.4	8.6 ± 0.2	93.7 ± 7
(D)							
IPSP and EPSP				IPSP_amp_	IPSP_dur_	EPSP_amp_	EPSP_dur_
CTRL				7.8 ± 0.6	92 ± 6	6.8 ± 0.5	47.5 ± 4.1
Cuprizone				8.2 ± 0.3	122.9 ± 11.7	2.8 ± 0.2	91.6 ± 11.9
7 days remyelination				9.8 ± 1.2	147 ± 30	6.5 ± 0.4	98.3 ± 12.7
25 days remyelination				8.5 ± 0.5	92.4 ± 6	7.1 ± 0.4	178.5 ± 18.4

Characteristics of membrane potential change after demyelination and remyelination at the resting membrane potential (RMP; A) and after continuous injection of a constant positive or negative current to hyperpolarize (−70 mV, B) or depolarize (−40 mV, C) the neuronal membrane as well as the amplitude and duration of EPSPs and IPSPs (D). Values represent mean ± SEM

*N* the number of cells, *THP* the threshold potential of action potentials, *APs* action potentials, *AHP* afterhyperpolarization, *amp* amplitude, *dur* duration

**Fig. 2:**
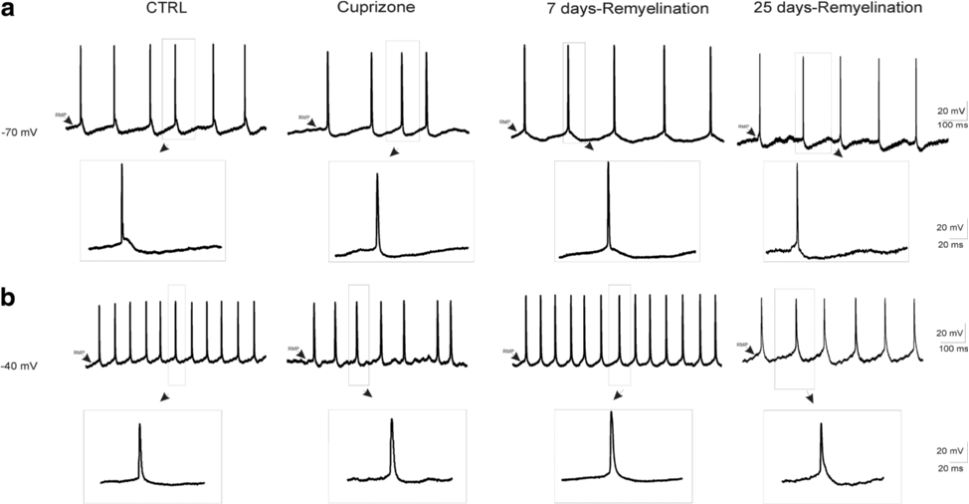
Intracellular injection of a constant positive or negative current was used to investigate how APs and AHP change at hyperpolarized (−70 mV) and depolarized (−40 mV) states of the membrane in neurons of layer 4 of the neocortical A1 area in control, cuprizone, 7 days remyelination, and 25 days remyelination mouse groups. Representative traces of the membrane potential deflections at −70 mV (**a**) and −40 mV (**b**) of neurons in different mouse groups are shown

Hyperpolarization of A1 neurons to −70 mV reduced spontaneous AP firing (Fig. 2a). Under control condition, the AP threshold was significantly larger compared to cuprizone as well as the early and late remyelination groups (*P* < 0.001; Fig. 2a, Table 1). Neurons recorded from slices after 25 days of remyelination exhibited a larger and longer AHP of APs when hyperpolarized to −70 mV compared to cuprizone and 7 days remyelination groups (*P* < 0.001; Fig. 2a, Table 1). There was no significant difference in the amplitude of APs between all groups when neurons were hyperpolarized to −70 mV (Fig. 2a, Table 1). However, hyperpolarization of neurons to −70 mV significantly decreased the duration of APs in slices from mice after 7 days of remyelination compared to other groups (Fig. 2a, Table 1).

When neurons were depolarized to −40 mV, spontaneous firing increased under all experimental conditions (Fig. 2b). Comparing the amplitude and duration of APs from neurons of slices prepared from control and cuprizone-treated animals revealed no significant difference (Fig. 2b, Table 1). However, the amplitude of APs in preparations obtained from 7 and 25 days remyelination mice was higher than APs of cuprizone-treated mice (*P* < 0.001; Fig. 2b, Table 1). Depolarization of neurons to −40 mV significantly increased the amplitude and duration of the AHP of APs, as well as the duration of APs, in slices after 25 days of remyelination compared to slices from cuprizone and 7 days remyelination mice (*P* < 0.001; Fig. 2b, Table 1).

**Fig. 3:**
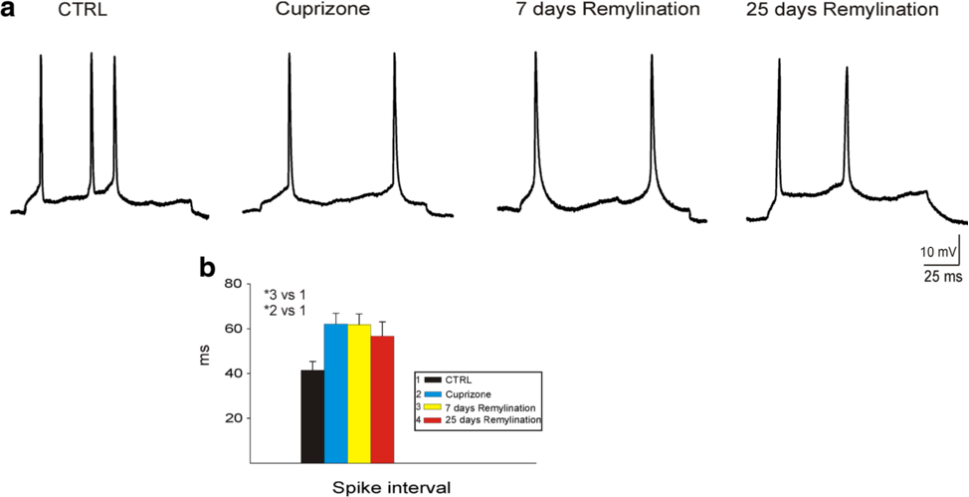
Typical discharge patterns of A1 cortical neurons from control, cuprizone, 7 days remyelination, and 25 days remyelination mice induced by intracellular injection of square positive current pulses (120 ms; 100 pA). **a** Representative traces of the discharge patterns induced at resting membrane potentials of −52 mV in different mice groups and a diagram of the thalamocortical slice, indicating recording site for intracellular recordings. **b** *Group of bars* represents the mean ± S.E.M. of the interspike intervals (between the first and the second APs) between different mouse groups. Note that the neurons from cuprizone-treated animals exhibited longer interspike intervals compared to control and 7 days remyelination mice. *P < 0.001

Next, AP firing was induced by applying depolarizing current steps through the microelectrode (120 ms; 100 pA). Neurons from cuprizone-treated and 7 days remyelination animals exhibited longer interspike intervals compared to control mice, indicating slower spiking (*P* < 0.001; Fig. 3a, b). There were no significant differences in the frequency of APs and the amplitude of depolarization step after current injection among different groups.

### The effect of de- and remyelination on EPSP and IPSP

**Fig. 4:**
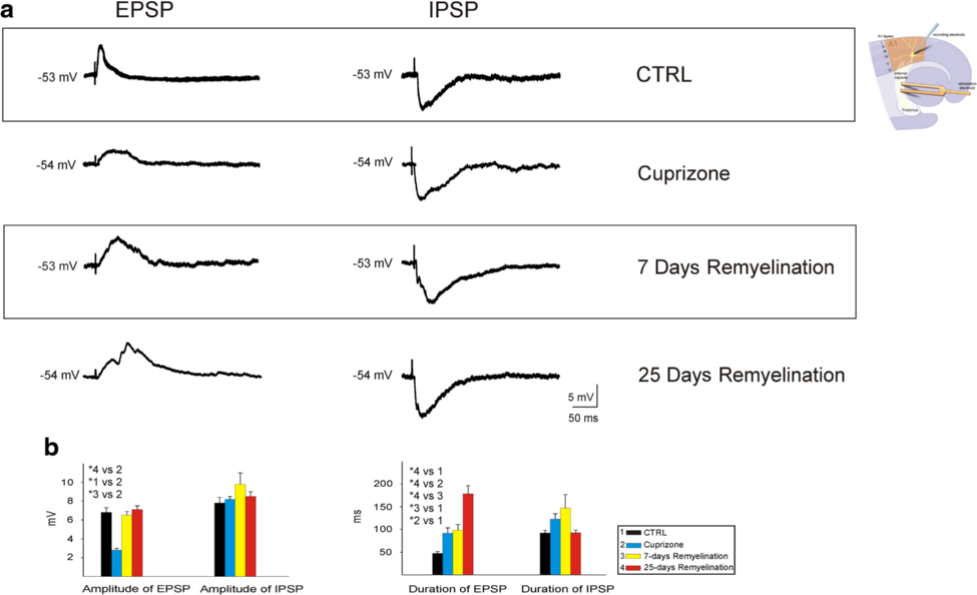
Excitatory and inhibitory postsynaptic potentials (EPSP and IPSP) recorded by neurons of layer 4 of A1 neocortex after stimulation of thalamocortical pathways (diagram) in control, cuprizone, 7 days remyelination, and 20 days remyelination mice. **a** Representative traces of EPSP and IPSP in different mice group. **b** *Group of bars* represents the mean ± S.E.M. of the amplitude and duration of EPSPs and IPSPs in different mouse groups. *P < 0.001

To study the characteristics of synaptic transmission, EPSPs and IPSPs were evoked by the stimulation of the thalamocortical projections in rostral position with respect to the cortex during intracellular recordings of A1 neurons (with ~50 % failure rate). The synaptic delay of IPSPs and EPSPs evoked by thalamocortical stimulation was about 3–4 ms. There were no significant differences in the amplitude and duration of IPSPs between different animal groups (Fig. 4a, b). However, the amplitude of EPSPs was significantly lower in cuprizone mice compared to other groups (*P* < 0.001; Fig. 4a, b). In addition, the duration of EPSPs was significantly longer in slices obtained from 25 days remyelination mice compared to the other groups (*P* < 0.001; Fig. 4a, b). The duration of EPSPs in control mice were also shorter than in the other groups (*P* < 0.001; Fig. 4a, b).

### The effect of de-and remyelination on LTP

**Fig. 5:**
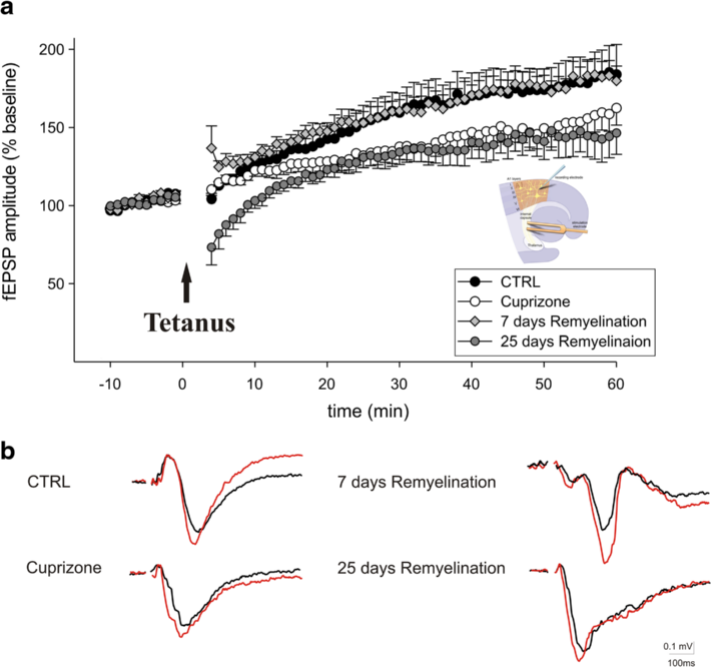
Long-term potentiation (LTP) of the evoked field excitatory postsynaptic potentials (fEPSPs) in the neocortical A1 area induced by a conditioning tetanic stimulation delivered to the thalamocortical pathway of combined neocortical slices (diagram). **a** Tetanic stimulation (four trains of 100-ms long train of 100-Hz electrical pulses, pulse duration 0.1 ms, interpulse interval 10 ms, train interval of 100 ms) produces a potentiation in the amplitude of the evoked field potentials, calculated as a percentage of baseline mean response amplitude. *Solid circles*, *open circles*, *solid square*, and *solid triangles* show the evoked fEPSP in control, cuprizone, 7 days remyelination, and 20 days remyelination mouse groups, respectively. *Arrow* points the time of tetanic stimulation. The time points given refer to LTP induction. Demyelination following cuprizone treatment significantly reduced LTP induction. During the early remyelination phase (7 days), LTP of fEPSP returned to control levels. However, the ratio of LTP decreased during the late (25 days) phase of remyelination. **b** Representative examples of the evoked field potentials before and after tetanic stimulation in different experimental groups. *Values* represent mean ± SEM

To assess possible changes in long-lasting synaptic plasticity, a conditioning tetanic stimulation was delivered to the internal capsule of thalamocortical slices after 30 min of stable recording and followed by pulses with stimulation parameters identical to control levels. Application of tetanic stimulation produced a stable and lasting enhancement of the amplitude of fEPSPs in all tested slices of control mice (*n* = 13, 163.6 ± 5.7 % control; Fig. 5a, b). Demyelination following cuprizone treatment significantly reduced LTP induction in A1 from all tested slices (*n* = 14, 137.2 ± 3.7 % baselines, *P* < 0.05, Fig. 5a, b). During the early remyelination phase, LTP recovered to a level of 161 ± 3.9 % of baseline which was significantly increased compared to the potentiation under control conditions (*n* = 10, *P* < 0.05, Fig. 5a, b). However, during the late phase of remyelination, the degree of LTP decreased to 135.6 ± 3.6 % of baseline (*n* = 15, *P* = 0.001, Fig. 5a, b).

### Histological evaluation of de- and remyelination in the cuprizone model

**Fig. 6:**
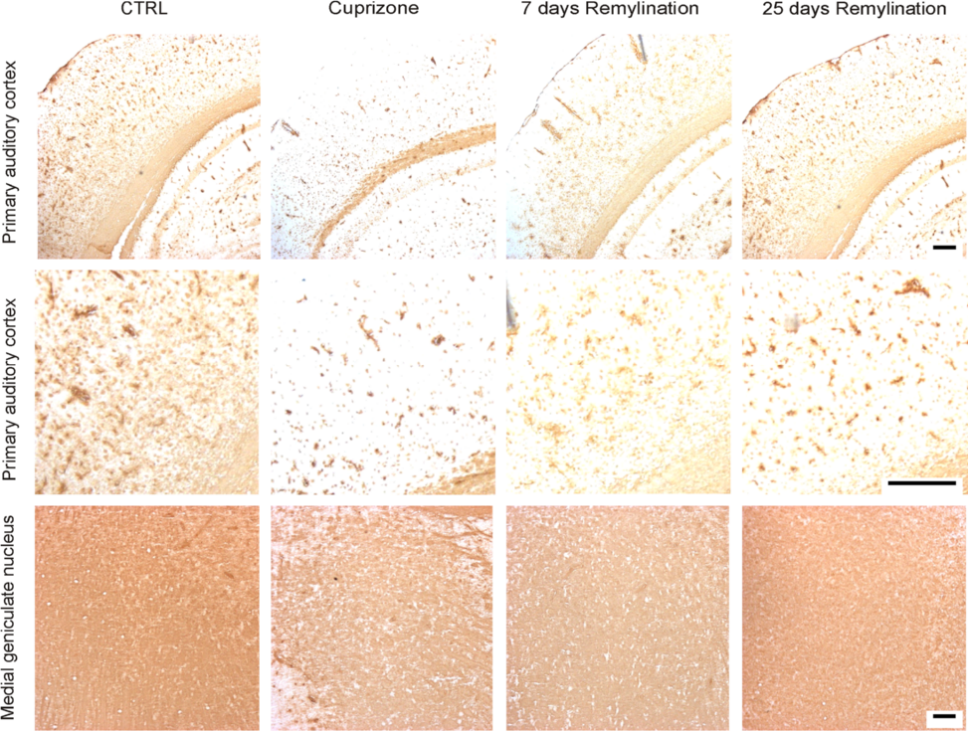
Representative micrographs of the primary auditory cortex and the medial geniculate nucleus in the control group, cuprizone group, and 7 days as well as 25 days remyelination groups. Treatment with the copper chelator cuprizone after 5–6 weeks induced demyelination in both the primary auditory cortex and the medial geniculate nucleus (cuprizone group). Termination of cuprizone treatment and administration of normal food allowed remyelination (7 days and 25 days remeylination groups)

Treatment with cuprizone is a well-established model of general demyelination usually occurring after 5–6 weeks of administration in mice. In line with previous studies [22, 27], myelin content was decreased of 83 % of the control value in the primary auditory cortex and 34 % in the medial geniculate nucleus, which are parts of a reciprocally connected network (Fig. 6). Termination of cuprizone treatment by administration of normal food allowed remyelination, which reach the maximal recovery after about 4 weeks [23, 24]. Indeed, myelin content after 25 days increased in the primary auditory cortex by 66 % compared to the cuprizone group. In the medial geniculate nucleus, myelin content returned to the control value in the same period (Fig. 6).

## Discussion

Our results revealed the impact of myelin integrity on firing properties and synaptic transmission of neurons in the auditory thalamocortical system. Myelin loss and restoration affected cellular activities in thalamorecipient layer 4 as well as cortical synaptic transmission in the third layer of the auditory cortex. In addition, the early and late phases of remyelination differentially changed neuronal excitability and synaptic plasticity of cuprizone-treated mice.

Myelination of the thalamic projections innervating the auditory cortex begins around 1 year of age and progresses until the fourth year [31]. The main thalamocortical input from the ventral part of the medial geniculate body of the thalamus is directed in a tonotopic manner to layer 4 of the A1 [32]. The information from cells in layer 4 of the auditory cortex project to the pyramidal neurons of layer 3, and from there, the inputs are distributed to the other cortical layers of both the ipsilateral and the contralateral auditory cortex through the corpus callosum. The corpus callosum, the largest fiber tract in the brain, is affected in most cases of MS patients as well as in cuprizone-treated mice [33, 34]. The isthmus of the corpus callosum contains fibers from the motor, the somatosensory, and the primary auditory cortices [34]. Our results revealed that demyelination altered thalamocortical inputs in primary auditory cortex, which might produce difficulty with sensory perception in MS [35]. Using diffusion tensor imaging fiber tracking, a tenfold higher density of lesions in thalamocortical projections compared to other brain white matter regions was observed [36].

Our data revealed a decreased excitability of layer 4 neurons from A1 in cuprizone-treated mice, characterized by more negative RMPs, longer evoked spiking intervals, and smaller EPSPs. Axonal demyelination increases stability of the membrane potential and leads to impaired neuronal function in demyelinating diseases by increasing the input capacitance and changing the surface exposure of ionic channels, especially K^+^ channels [37]. In the present study, layer 4 neurons revealed a significant hyperpolarization of the RMP after cuprizone treatment. Intracellular recording from A1 neurons has shown that depolarization and hyperpolarization of the membrane potential underlie excitatory-inhibitory response properties to thalamic inputs [38]. Alterations of depolarizing shifts have been also reported in the membrane potential of peripheral nerves when recordings were performed near the site of demyelination [5]. In contrast to layer 4 neurons in our study, demyelination of the axon of layer 5 neurons in the auditory cortex causes spontaneous intrinsic and network excitability of pyramidal neurons of cuprizone-treated mice [7]. In addition to the well-known effect of layer 4 neurons to drive feed-forward and recurrent inhibition in other cortical layers [39, 40], it has been shown that enhanced layer 4 activity directly suppressed layer 5 neurons by activating deep, fast-spiking inhibitory neurons in awake, behaving mice [41]. A pronounced reduction in layer 4 activity by hyperpolarization of neurons enhanced layer 5 firing and broadened the representation of horizontal space across the population of layer 5 regular spiking neurons [41]. Hyperpolarization and decreased excitability of layer 4 neurons observed in cuprizone-treated mice in our study caused disinhibition and thereby increased excitability in other cortical layers. Disturbed spatiotemporal connections between different areas of cortical networks have been suggested to underlie cognitive impairment, commonly observed in MS patients [41, 42]. Damage to heterogeneous profiles of myelination of different neocortical layers may disorganize different arrays of inter-layer communication and prevent the emergence of complex cellular behaviors [43].

In general, it has been shown that the spatiotemporal patterns of de- and remyelination vary across the brain after 6 weeks of cuprizone administration and subsequent remyelination. This suggests varying susceptibility to injury and/or ability to repair in the brain in the cuprizone mouse model [44]. Therefore, partially diversified results may be expected. Following cessation of a 6-week cuprizone diet, demyelinated lesions demonstrate about 50 % recovery after 1 week and an almost complete remyelination was observed within 4 weeks [45]. Our data indicate a partial recovery of neuronal excitability and synaptic plasticity in the early and late phases of remyelination. In keeping with our results, it has been shown that while immunohistochemical staining points to extensive remyelination, neurotransmission along previously demyelinated neuronal tissue has not been completely recovered to normal [46]. Following 6 weeks of cuprizone ingestion, the recovery of action potentials was incomplete in an ex vivo slice preparations model even after feeding with normal chow for another 6 weeks [47]. In a similar way, cuprizone feeding increased the response latency between the left and right sensorimotor cortices and only partial recovery of axonal conduction was observed after remyelination [48]. Early periods of remyelination, prior to new myelin formation, are associated with a redistribution and reaggregation of sodium channels close to the lesion site, a process that may render neurons more susceptible to injury [49]. It has been shown that production of de novo synapses with recruited oligodendrocyte progenitor cells by demyelinated bioelectrically active axons and release of glutamate are crucial for remyelination and recovery of lost function [50]. Different subtypes of sodium channel that are expressed during remyelination, as well as the effect of inflammatory mediators on its function, may be responsible for alterations of neuronal properties in the early and late phases of myelin restoration [51].

In our study, EPSPs evoked by stimulation of the thalamocortical projections in the control group were mainly monosynaptic in its early part and polysynaptic in its late part. The late components appeared with a smooth time course since the velocity of transmission in the pathways involved was not very different. Cuprizone-induced demyelination flattened the EPSPs as expected for demyelination. The late EPSPs became multiphasic during the remyelination process after both 7 and 25 days after withdrawal of cuprizone. It can be assumed that this is due to the alteration of the synaptic network which followed alterations in axonal conduction velocities on the basis of partial and/or patchy remyelination, thus leading to prolonged multiphasic EPSPs [52].

Our data indicated that demyelination reduced the amplitude of EPSPs and decreased synaptic plasticity at cortical synapses in the auditory cortex. Remyelination after 7 days restored the synaptic strength in these synapses. However, further remyelination was associated with impairment of LTP. In vivo intracellular recordings of the primary auditory cortex have shown that modulation of synaptic plasticity in this region is a prominent feature of synaptic responses to auditory stimuli [53]. These observations are in keeping with our results that LTP in the hippocampal CA1 area was decreased and LTP-related spatial memory was impaired in animal models of MS [54, 55]. Conversely, some other studies found no differences in hippocampal LTP between demyelinated and normal neuronal network [56]. Clinical studies indicate that the degree of neuroinflammation is associated with changes of LTP in MS patients [10]. It has been suggested that synaptic plasticity may be enhanced or impaired during experimental and clinical demyelination-remeylination, depending on the degree of neuroinflammation and the time point at which it is investigated [57].

## Conclusions

Our data indicate altered neuronal and synaptic activities between the thalamus and auditory cortex in the cuprizone model of demyelination/remyelination. Demyelination and remyelination differentially affect the characteristic features of neuronal activities and synaptic plasticity in the thalamocortical auditory system. Further studies are needed to clarify whether these alterations reflect the degree of neuroinflammation as well as the extent of clinical manifestations of MS.

## Abbreviations

AHP, afterhyperpolarizations; APs, action potentials; EPSPs, excitatory postsynaptic potentials; fEPSPs, field excitatory postsynaptic potentials; IPSPs, inhibitory postsynaptic potentials; LTP, long-term potentiation; MS, multiple sclerosis; PFA, paraformaldehyde; PLP, myelin proteolipid protein; RMP, resting membrane potential; THP, the threshold potential of action potentials
